# MicroRNA-147b Regulates Vascular Endothelial Barrier Function by Targeting ADAM15 Expression

**DOI:** 10.1371/journal.pone.0110286

**Published:** 2014-10-15

**Authors:** Victor Chatterjee, Richard S. Beard, Jason J. Reynolds, Ricci Haines, Mingzhang Guo, Matthew Rubin, Jenny Guido, Mack H. Wu, Sarah Y. Yuan

**Affiliations:** 1 Department of Molecular Pharmacology and Physiology, University of South Florida Morsani College of Medicine, Tampa, Florida, United States of America; 2 Department of Surgery, University of South Florida Morsani College of Medicine, Tampa, Florida, United States of America; 3 Department of Surgery, University of California Davis School of Medicine, Sacramento, California, United States of America; University of Kentucky, United States of America

## Abstract

A disintegrin and metalloproteinase15 (ADAM15) has been shown to be upregulated and mediate endothelial hyperpermeability during inflammation and sepsis. This molecule contains multiple functional domains with the ability to modulate diverse cellular processes including cell adhesion, extracellular matrix degradation, and ectodomain shedding of transmembrane proteins. These characteristics make ADAM15 an attractive therapeutic target in various diseases. The lack of pharmacological inhibitors specific to ADAM15 prompted our efforts to identify biological or molecular tools to alter its expression for further studying its function and therapeutic implications. The goal of this study was to determine if ADAM15-targeting microRNAs altered ADAM15-induced endothelial barrier dysfunction during septic challenge by bacterial lipopolysaccharide (LPS). An *in silico* analysis followed by luciferase reporter assay in human vascular endothelial cells identified miR-147b with the ability to target the 3′ UTR of ADAM15. Transfection with a miR-147b mimic led to decreased total, as well as cell surface expression of ADAM15 in endothelial cells, while miR-147b antagomir produced an opposite effect. Functionally, LPS-induced endothelial barrier dysfunction, evidenced by a reduction in transendothelial electric resistance and increase in albumin flux across endothelial monolayers, was attenuated in cells treated with miR-147b mimics. In contrast, miR-147b antagomir exerted a permeability-increasing effect in vascular endothelial cells similar to that caused by LPS. Taken together, these data suggest the potential role of miR147b in regulating endothelial barrier function by targeting ADAM15 expression.

## Introduction

ADAM15 is composed of five extracellular domains (prodomain, metalloprotease, disintegrin, cysteine-rich and EGF-like domains), and a cytoplasmic tail containing Src-homology docking sites [1]. This multi-domain structure exerts diverse functions in various biological or physiological processes [2]. The expression of ADAM15 in the vascular endothelium was first identified in 1997 [3]. Subsequent studies demonstrate its dual-function in proteolytic shedding of transmembrane molecules (thus named sheddase) and in regulating cell-cell/matrix adhesion and signaling [4]–[6]. More recently, studies show that ADAM15 supports cancer metastasis by promoting cell migration and angiogenesis [7], [8]. Also, increased ADAM15 is detected in atherosclerotic lesions, rheumatoid synovium, angiogenic retina, and intestines of patients with inflammatory bowel disease [9].

Given the lack of specific pharmacological inhibitors, research efforts have been devoted to generating and testing genetically modified mice. Interestingly, unlike ADAM10 or ADAM17 knockout, which causes embryonic lethality, ADAM15 knockout is viable and fertile but displays altered responses to insults (e.g., reduced angiogenesis to ischemia or hypoxia) [10]. Because targeting ADAM15 may block specific pathological responses with minimal side effects on basal physiological functions, it represents a promising area of interventional development [11].

Recently, we have reported that ADAM15 contributes to vascular endothelial hyperpermeability and promotes neutrophil and monocyte transendothelial migration in response to inflammatory stimulation [12], [13]. The signaling mechanisms underlying its barrier opening effect involve Src-mediated tyrosine phosphorylation and dissociation of endothelial junction molecules, such as VE-cadherin and γ-catenin [12], [13]. In a most recent study, we detected a significant increase of ADAM15 expression at both the gene and protein levels in mouse lungs following septic injury induced by bacterial lipopolysaccharide (LPS) injection; this effect was coupled with pulmonary edema and neutrophil infiltration. Interestingly, the LPS-induced endothelial barrier injury was greatly attenuated in ADAM15 knockout mice. [14]. Since increased ADAM15 expression contributes to endothelial barrier dysfunction during sepsis and inflammation, we sought to explore the therapeutic potential of suppressing ADAM15 expression in treating edematous injury. The goal of this study was to test the effects of microRNA-147b on ADAM15-induced endothelial hyperpermeability during septic challenge.

MicroRNAs (miRs) are small, noncoding RNAs that regulate gene expression and a variety of biological processes, including cell cycle, differentiation, development, and metabolism [15]–[17]. Accordingly, they have been implicated in disease states such as diabetes, immune or neurodegenerative disorders, and cancer [18]–[20]. As most miRs effect by repressing genes, miR mimics have become promising therapies against cancer or diseases involving gene/protein upregulation. [21]–[23]. Low molecular weight miR mimics can be effectively delivered as therapeutic agents in the form of double stranded oligonucleotides in a lipid based carrier vehicle [21], [24] or in viral vectors as used in traditional gene therapy [25], [26].

Considering the significant role of ADAM15 upregulation in LPS-induced endothelial hyperpermeability [14], we hypothesized that miRs that suppress ADAM15 might improve endothelial barrier integrity during LPS challenge. Our *in silico* analysis indicated miR-147b as a potential negative regulator of ADAM15 based on its ability to bind the 3′ UTR of ADAM15 mRNA in endothelial cells. Subsequently, we found that miR-147b mimic decreased ADAM15 expression and attenuated LPS-induced barrier dysfunction in endothelial cells. These findings suggest that ADAM15-targeting miR-147b may serve as an endothelial barrier protector against endotoxin-mediated inflammation.

## Materials and Methods

### *In Silico* Analysis of miRs Targeting ADAM15 3′ UTR

TargetScanS (http://www.targetscan.org), miRanda (http://www.microrna.org), and PicTar (http://www.pictar.org) prediction algorithms were used to identify candidate miR that target the 3′ UTR of ADAM15 gene. Based on the pairing of seed regions and calculated binding affinity, we selected miR-147b (GUGUGCGGAAAUGCUUCUGCUA) as a promising candidate miR for further testing.

### Luciferase assay

PmirGLO Dual-Luciferase miRNA Target Expression Vector (Cat. # E1330, Promega Corporation, Madison, WI, U.S.A.) was used to quantitatively evaluate miR activity by the insertion of ADAM15 3′ UTR immediately downstream of the firefly luciferase gene (luc2). Firefly luciferase is the primary reporter gene; reduced firefly luciferase expression indicates the binding of endogenous or introduced miR to the ADAM15 3′ UTR. This vector is based on Promega dual-luciferase technology, with firefly luciferase (luc2) used as the primary reporter to monitor mRNA regulation and Renilla luciferase (hRluc-neo) acting as a control reporter for normalization and selection. 48 hours (h) after co-transfection with the pmirGLO/ADAM15/3′ UTR vector constructs and miR-147b mimic, endothelial cells were analyzed for luciferase activity using the Dual-Glo Luciferase Assay System (Cat. # E2920, Promega Corporation, Madison, WI, U.S.A.) and a MicroLumatPlus LB96V luminometer (Berthold Technologies, Oak Ridge, TN, U.S.A.). Normalized firefly luciferase activity (firefly luciferase activity/Renilla luciferase activity) for each construct was compared to that of the pmirGLO Vector no-insert control. For each transfection, luciferase activity was averaged from six replicates.

### Western Blotting

Human lung microvascular endothelial cells (HLMECs) (Cat. # H-6011, Cell Biologics Inc., 2201 West Campbell Drive, Chicago, IL, U.S.A.) and human umbilical vein endothelial cells (HUVECs) (Cat. # C2517A, Lonza Walkersville, Inc. Walkersville, MD, U.S.A.) were transfected with miR-147b mimic, control miR, or their respective inhibitors as described below and grown to confluence in 6 well plates. Endothelial cell lysates were prepared after 48 h of transfection by harvesting cells in 100 µl cold 1× RIPA extraction buffer (Cat. # R0278, Sigma Aldrich, St. Louis, MO, U.S.A.) containing protease inhibitors (Cat. # 05892970001, Roche Diagnostics, Indianapolis, IN, U.S.A.). Cell lysates were run on a SDS-PAGE electrophoresis gel and transferred onto a nitrocellulose membrane. Membrane was blocked with Odyssey blocking buffer (Cat. # 927-40000, Li-Cor Biosciences, Lincoln, Nebraska, U.S.A.) for 1 h at room temperature followed by overnight probing with rabbit anti-human ADAM15 N-terminal antibody (dil 1∶1000, Cat. # ab137387, Abcam, Cambridge, MA, U.S.A.) at 4°C. After overnight incubation with primary antibody, the membrane was washed in 1× PBS with 0.1% Tween 20 for 30 minutes with change of washes every 5 minutes. This was followed by secondary antibody incubation with donkey anti-rabbit IRDye 800 Licor secondary (dil 1∶20,000, Cat. # 926-32213, Li-Cor Biosciences) for 1 h at room temperature. The membrane was washed in 1× PBS with 0.1% Tween 20 for 30 minutes with change of washes every 5 minutes and then imaged in an Odyssey CLx infrared imaging system.

### Immunofluorescence microscopy

HUVECs were transfected with scrambled miR or miR-147b mimic and plated on gelatin- coated coverslips. 48 h after transfection, cells were fixed in 4% paraformaldehyde for 10 minutes at room temperature followed by repeated washes with 1× PBS. After blocking with 5% BSA for 1 h at room temperature, cells were incubated with anti-human ADAM15 (dil 1∶100, Cat. # ab137387, Abcam, Cambridge, MA, U.S.A.) overnight at 4°C followed by secondary donkey anti-rabbit Alexa Fluor 488 (dil 1∶200, Cat. # A-21206, Life Technologies) for 1 h at room temperature. Cells were observed under a Leica confocal microscope.

### Flow Cytometry

HUVECs were detached with enzyme-free cell dissociation solution (Cat. # S-004-C, EMD Millipore, Billerica, MA, U.S.A.). To examine the surface expression of ADAM15, transfected HUVECs were stained with anti-ADAM15 ectodomain antibody (Cat. # MAB935, R&D Systems, Minneapolis, MN, U.S.A.) for 30 min on ice followed by incubation with Cy3-conjugated secondary antibody (Cat. # 715-166-150, Jackson ImmunoResearch Laboratories, West Grove, PA, U.S.A.) for another 30 min. In all experiments, an identical amount of isotype IgG (Cat. # MAB002, R&D Systems) was applied as a control for nonspecific staining.

### Transfection of miR-147b mimic and antagomir

Using a Nucleofector 2b electroporation device, HUVECs and HLMECs were transfected by electroporation with 200 nM miR-147b mimic (Cat. # MC 12874, Life Technologies) or miR negative control (Cat. # AM17110, Life Technologies) in 100 µL of Amaxa nucleofector solution (Cat. # VPB 1002 & VPB 1003) per reaction. In separate groups, HUVECs and HLMECs were transfected with 200 nM anti-miR-147b (Cat. # MH12874, Life Technologies) or miR inhibitor negative control (Cat. # AM17010, Life Technologies). Cells were then assayed for ADAM15 expression and monolayer permeability at 48 h after miR mimic or antagomir transfection.

### Endothelial Barrier Resistance

HUVECs were seeded onto Electric Cell-Substrate Impedance Sensing (ECIS) 8W10E+ PET electrode arrays (Applied Biophysics, Troy, NY, U.S.A.) and grown to confluence for quantitative analyses of transendothelial electric resistance (TER). Resistance was measured 48 h post-transfection with and without treatment with 200 ng/ml LPS for 24 h. TER values are reported as resistance normalized to the respective controls.

### Endothelial Monolayer Permeability Assay

HUVECs and HLMECs were grown to confluence on Corning Co-Star transwell membrane (Cat. # 3413, Corning Inc. Tewksbury, MA 01876, U.S.A.) (pore size 0.4 µm). 48 h after transfection, cells were treated with 200 ng/ml LPS (Cat. # 4391, Sigma Aldrich, St. Louis, MO, U.S.A.) for 24 h. Then 0.5 mg/ml of fluorescein isothiocyanate (FITC) conjugated albumin (Cat. # A-9771, Sigma Aldrich) was added to the top chamber, and albumin transendothelial flux was measured using a BioTek fluorescence microplate reader after 1 h of equilibration. The permeability coefficient of albumin (*Pa*) was calculated as *Pa*  =  [*27*] */t* ×1/*A* × *V*/*[L]*, where *[A]* is the bottom chamber concentration, *t* the time (s), *A* the area of the membrane (cm^2^), *V* the bottom chamber volume, and *[L]* the top chamber concentration.

### Statistical analysis

An unpaired Student's *t*-test was used to compare differences between two groups. ANOVA was used followed by Newman-Keuls post hoc for comparison of multiple groups. Statistical significance was defined as *P*≤0.05.

## Results

### miR-147b specifically binds the 3′ UTR of ADAM15

**Figure 1:**
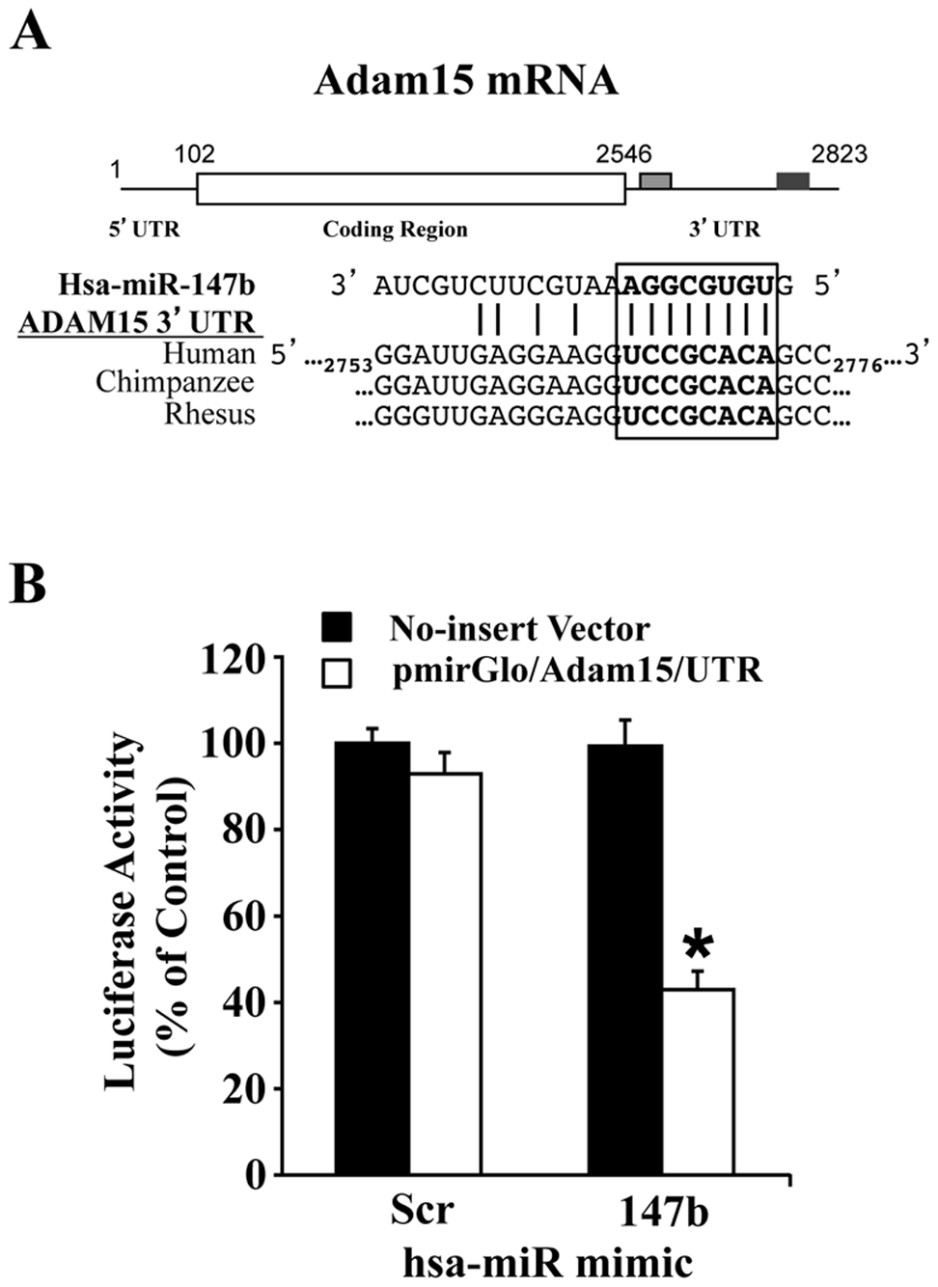
miR-147b targets ADAM15 3′ UTR. (A) Schematic illustration of ADAM15 mRNA and sequence alignments between ADAM15 3′ UTR and hsa-miR-147b. (B) HUVECs were co-transfected with a miR mimic and pmirGlo/ADAM15 containing ADAM15 3′ UTR downstream of the luciferase reporter. Cells were analyzed for luciferase activity 48 h post-transfection. Data represent mean ± SEM. *P<0.05 vs. scrambled control.

We first sought to identify specific miRs that can directly target ADAM15 mRNA. An *in silico* analysis confirmed that miR-147b displayed high complementarity and binding specificities with putative binding sites in ADAM15 3′ UTR as shown in Fig. 1A. We next confirmed the specific targeting of miR-147b to ADAM15 mRNA using a dual-Glo luciferase reporter construct (pmirGlo/ADAM15/UTR) with ADAM15 3′ UTR inserted immediately downstream of the luciferase reporter gene. HUVECs were co-transfected with this construct or an empty (no insert) vector and miR-147b mimic or a scrambled miR and expression of luciferase activity was quantitated after 48 h. Upon co-transfection with scrambled miR and pmirGlo/ADAM15/UTR or empty vector, significant luciferase activity was observed, however co-transfection with miR-147b mimic significantly attenuated luciferase activity only in pmirGlo/ADAM15/UTR transfected cells, supporting that miR-147b binding to the ADAM15 3′ UTR is specific (Fig. 1 B).

### miR-147b downregulates ADAM15

**Figure 2:**
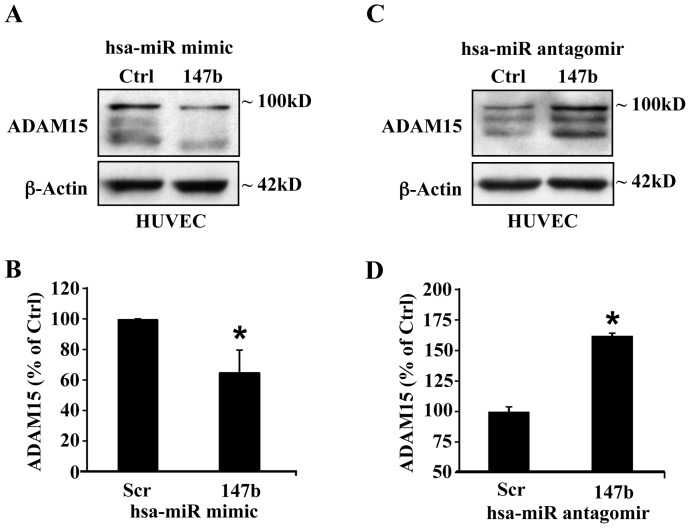
miR-147b down-regulates ADAM15 expression in endothelial cells. (A) Representative Western blot from HUVECs transfected with 200 nM miR-147b mimic or a scrambled miR control. (B) Quantitative densitometric analysis of ADAM15 expression at 48 h post-transfection. (C) Representative Western blot from HUVECs transfected with 200 nM miR-147b antagomir or a control antagomir. (D) Quantitative densitometric analysis of ADAM15 expression at 48 h post-transfection. Data represent mean ± SEM. *P<0.05 vs. scrambled control.

To assess the impact of miR-147b on ADAM15 expression in endothelial cells, we transfected hsa-miR-147b in HUVECs and compared ADAM15 expression between cells transfected with miR-147b and miR control with scrambled sequence. As shown in Fig. 2 A&B, transfection of hsa-miR-147b significantly decreased ADAM15 protein expression in HUVECs compared to transfection with scrambled sequence miR. Conversely, transfection with miR-147b antagomir increased ADAM15 expression in HUVECs as compared to control miR antagomir (Fig. 2 C&D).

**Figure 3:**
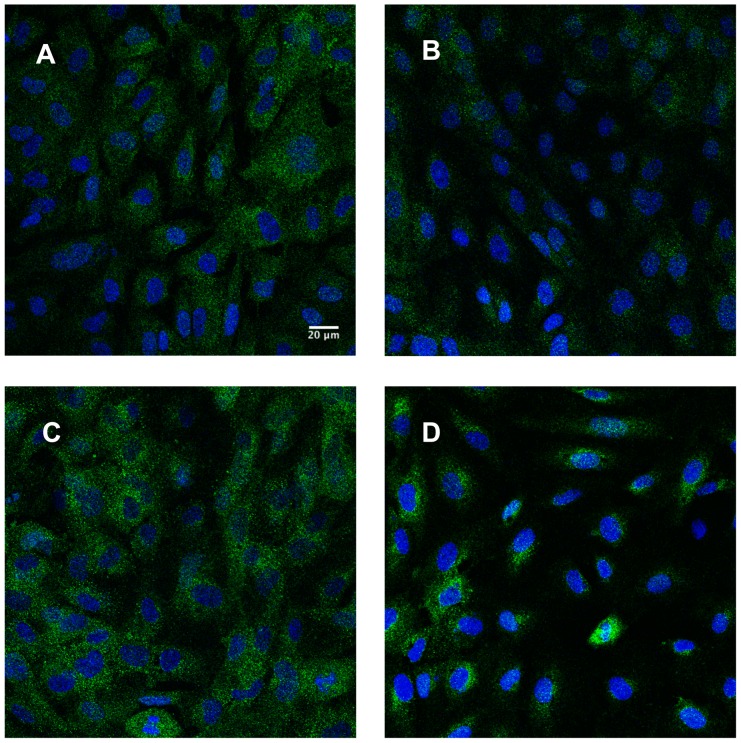
miR-147b attenuates LPS-induced increase in ADAM15 expression. Immunofluorescence labeling of ADAM15 in HUVECs transfected with scrambled miR (A), miR-147b mimic (B), scrambled miR and 200 ng/ml LPS (C), and miR-147b mimic and 200 ng/ml LPS (D). Green denotes ADAM15 staining, blue is DAPI staining of nuclei. Scale in (A) corresponds to 20 µm and applies to all images.

We also examined the effect of miR-147b on ADAM15 expression in HUVECs using immunofluorescence microscopy. The level and overall intensity of ADAM15 staining was lower in miR-147b transfected cells compared to scrambled miR transfected cells (Fig. 3 A&B). After 200 ng/ml LPS administration for 24 h, ADAM15 expression levels increased in scrambled miR transfected cells. However, this effect was not observed in cells transfected with miR-147b mimic (Fig. 3 C&D).

**Figure 4:**
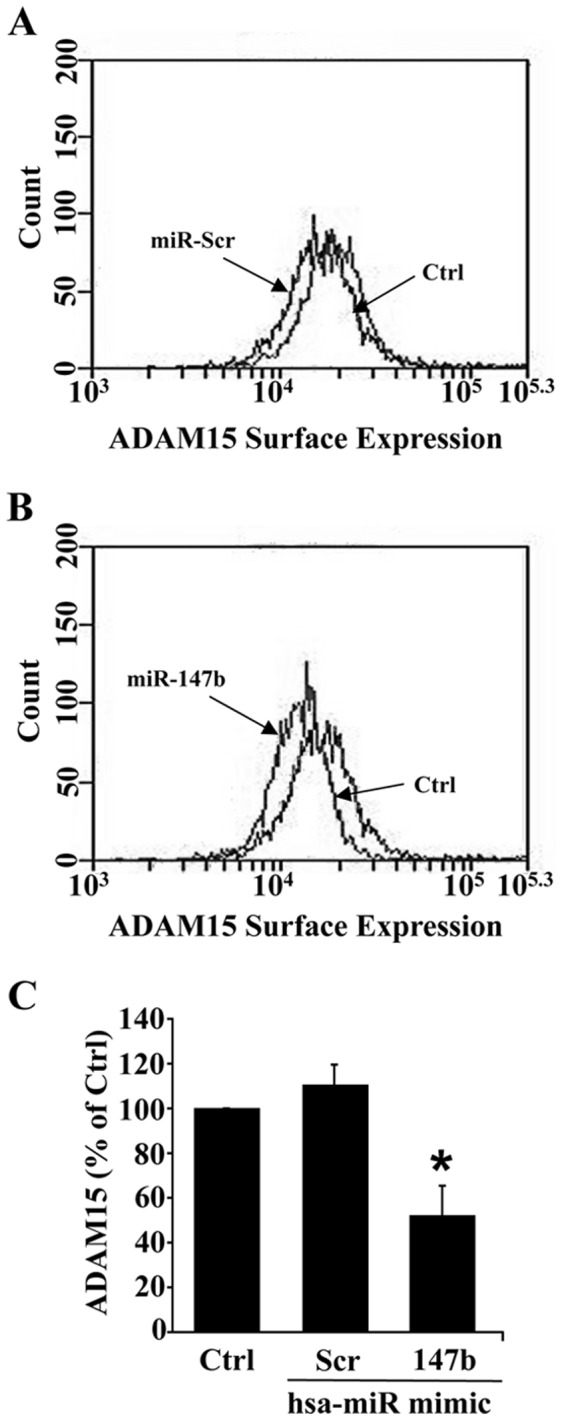
miR-147b down-regulates cell surface expression of ADAM15 in endothelial cells. At 48 h after transfection, live HUVECs were labeled with a monoclonal anti-ADAM15 ectodomain antibody and a secondary Cy3 conjugate. Cell surface expression was analyzed by flow cytometry (A–B). The bar graph shows quantitative cell surface ADAM15 expression in different treatment groups, each derived from 3 separate experiments (C). Data represent mean ± SEM. * *P*<0.05 vs. untreated control.

Next, we investigated whether miR-147b mimic transfection decreased cell surface expression of ADAM15 by using FACS labeling with an antibody directed against the ectodomain of ADAM15 in live HUVECs. As shown in Fig. 4, we found that hsa-miR-147b transfection decreased cell surface expression of ADAM15 as compared to untreated controls. There was no difference in cell surface ADAM15 expression between untreated control and scrambled miR transfected HUVECs.

### miR-147b improves basal endothelial barrier function

**Figure 5:**
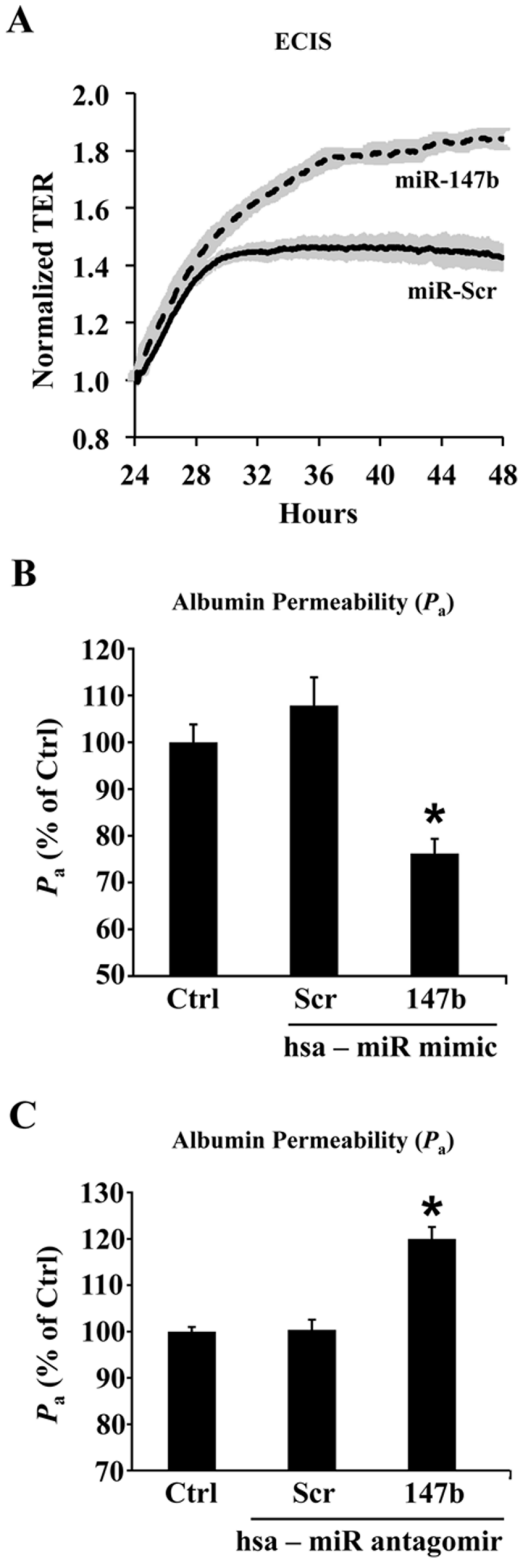
miR-147b decreases endothelial permeability. (A) In HUVECs, transfection with miR-147b mimic enhanced endothelial barrier function as indicated by increased transendothelial electric resistance (TER) compared to scrambled miR transfection. (B) Transfection with miR-147b mimic decreased albumin flux across HUVEC monolayers (*Pa*) measured 48 h post-transfection. *P<0.05 vs. untreated control. (C) Transfection with miR-147b antagomir increased endothelial monolayer permeability to albumin measured 48 h post-transfection. Data represent mean ± SEM. *P<0.05 vs. untreated control.

Previous studies have shown that ADAM15 overexpression caused increased endothelial cell permeability in both unstimulated and inflammatory mediator-stimulated conditions [13]. This study further tested whether the regulatory effect of miR-147b on ADAM15 expression had any functional consequences on the endothelial permeability response. As indicators of endothelial barrier function, transendothelial electric resistance and transendothelial flux of albumin were measured. Transfection of HUVEC monolayers with miR-147b mimic elevated TER, indicating enhanced barrier function (Fig. 5A). Similarly, transfection of hsa-miR-147b significantly decreased basal endothelial permeability to albumin compared to scrambled miR (Fig. 5B). To study the functional significance of inhibition of endogenous miR-147b on endothelial barrier function, we transfected HUVECs with antagomirs of miR-147b and a control miR antagomir for 48 h and measured endothelial monolayer permeability to FITC-albumin. Transfection of miR-147b antagomir, but not the control antagomir, induced a significant increase in endothelial permeability to albumin as shown in Fig. 5C. This suggests that inhibition of endogenous miR that target ADAM15 may increase endothelial barrier dysfunction by upregulation of ADAM15 expression in HUVECs.

### LPS-induced endothelial barrier dysfunction is attenuated by miR-147b

**Figure 6:**
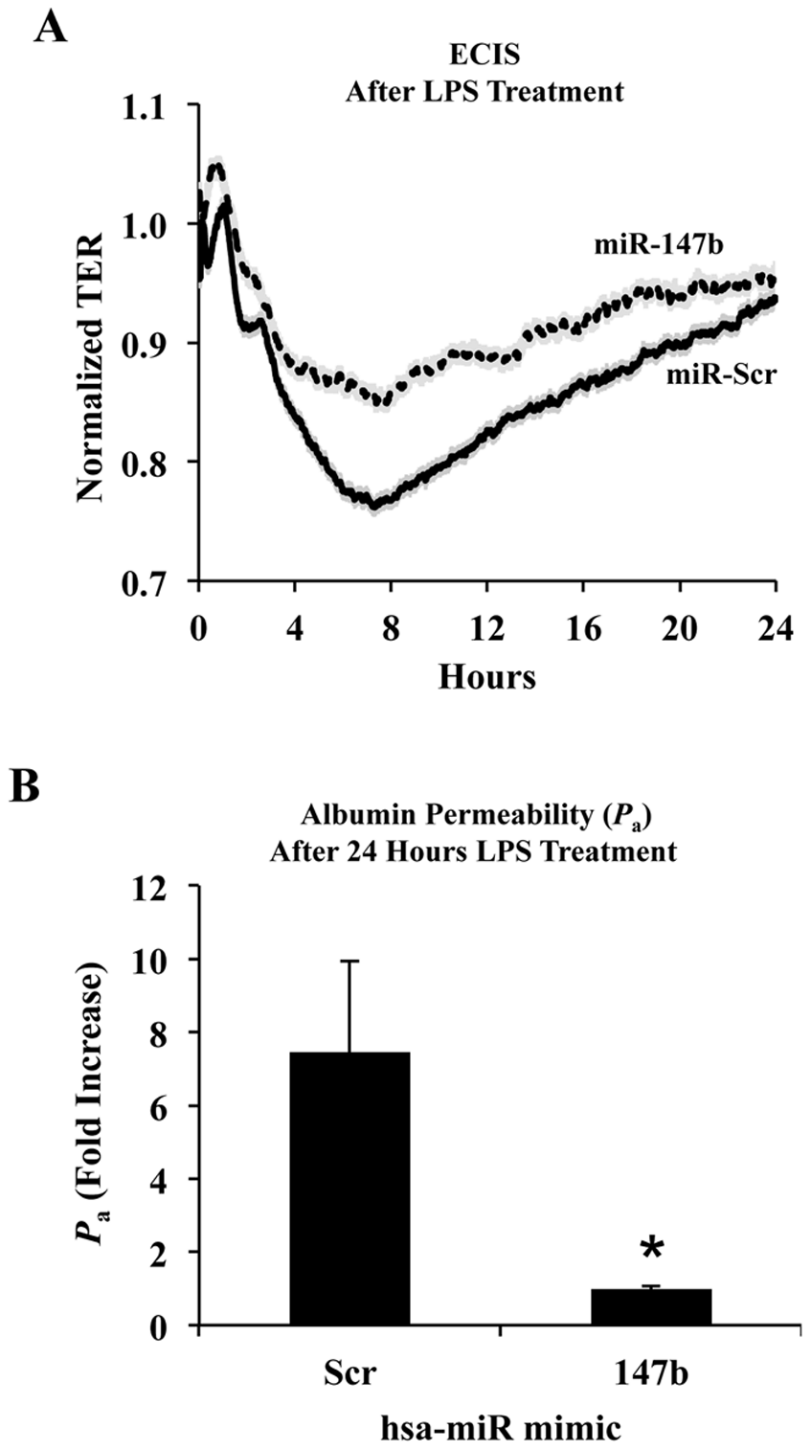
miR-147b attenuates LPS-induced endothelial hyperpermeability. (A) Transfection with miR-147b mimic reduced the drop in transendothelial barrier resistance caused by LPS treatment. (B) Transfection with miR-147b mimic decreased albumin permeability across HUVEC monolayers after LPS treatment. Data represent mean ± SEM. *P<0.05 vs. scrambled miR.

In order to determine whether miR-147b alters LPS-mediated barrier dysfunction, we measured TER in hsa-miR-147b and control miR transfected HUVECs with or without LPS treatment using an ECIS system. As shown in Fig. 6A, the decrease in resistance following LPS treatment for 24 h was significantly attenuated in miR-147b transfected cells as compared to scrambled miR-treated cells. Next, we investigated whether hsa-miR-147b transfection in HUVECs could attenuate LPS induced endothelial permeability to albumin. As shown in Fig. 6B, hsa-miR-147b transfected HUVECs showed significantly decreased permeability to albumin after LPS treatment compared to scrambled miR. These results suggest that miR-147b protects endothelial barrier function during LPS challenge.

### miR-147b improves human lung endothelial barrier function by down-regulating ADAM15 expression

**Figure 7:**
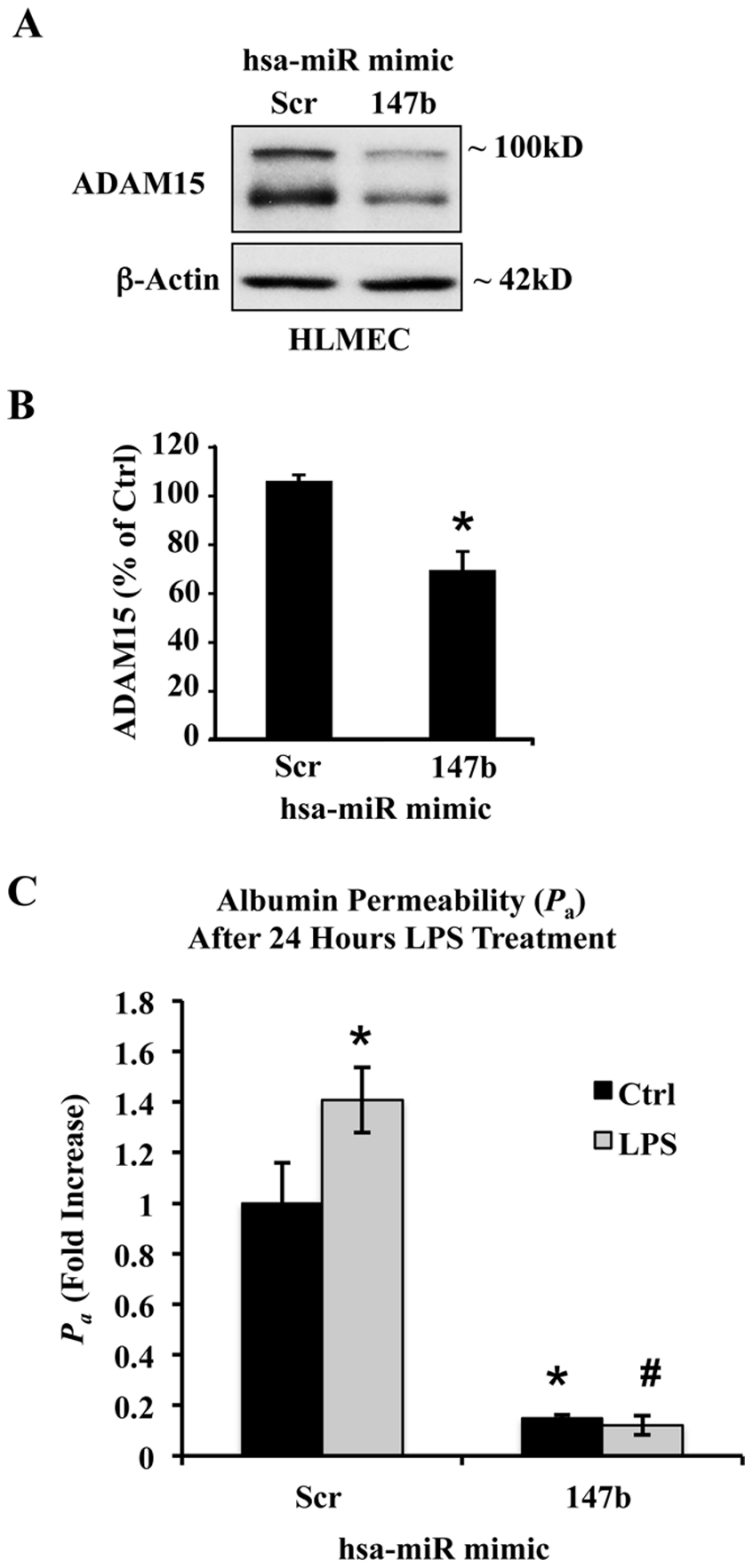
miR-147b attenuates LPS-induced endothelial hyperpermeability to albumin in HLMECs by targeting ADAM15. (A) Representative Western blot from human lung microvascular endothelial cells (HLMEC) transfected with 200 nM miR-147b mimic or a miR negative control. (B) ADAM15 expression was measured 48 h post-transfection and quantified with densitometry. (C) Incubation with 200 ng/ml LPS for 24 h caused an increase in albumin permeability across HLMEC monolayers, an effect diminished in cells transfected with miR-147b. Data represent mean ± SEM. *P<0.05 vs. control scrambled miR, ^#^ *P*<0.05 vs. LPS scrambled miR.

To confirm that the effects of miR-147b on ADAM15 expression and barrier function were not unique to HUVECs, we repeated the same experiments in human lung microvascular endothelial cells (HLMECs). As shown in Fig. 7A, miR-147b transfection significantly decreased ADAM15 expression in HLMECs to the same extent as seen in HUVECs. Similarly, HLMEC transfection with miR-147b attenuated LPS-induced endothelial hyperpermeability to albumin as compared to scrambled miR-transfected cells (Fig. 7B). This suggests that miR-147b rescues the barrier dysfunction in response to LPS injury in endothelial cells of different origins.

## Discussion

Systemic inflammatory injury is a major cause of mortality and morbidity in patients with sepsis, which affects 750,000 Americans annually costing $17 billion for hospital care [28]–[31]. Despite the advanced theories regarding its pathogenesis, there has been no major breakthrough in treatment or prevention. The limited clinical progress is partially attributed to the underdevelopment of effective therapies, owing to the complexity of the disease and incomplete understanding of its endpoint cellular mechanisms [27], [32]. Microvascular disorders represent a common endpoint of inflammatory response to various types of injury [33]–[36], where endothelial barrier dysfunction causes fluid leakage and leukocyte infiltration in vital organs, especially in the lungs, leading to respiratory distress and multiple organ failure [37]–[41]. The essential role of endothelial barrier dysfunction in inflammatory injury highlights the need to develop target-directed treatments [42], [43].

ADAM15 is a pro-inflammatory molecule with its expression and function upregulated in various tissues during the development of metastatic cancer and autoimmune diseases [7], [44]–[46]. Recently, we have identified a novel function of ADAM15 in mediating endothelial barrier dysfunction during inflammatory injury [12], [14]. ADAM15 was upregulated in the lung endothelium of septic mice, and ADAM15 gene knockout significantly attenuated lung inflammation and pulmonary edema caused by LPS administration [14]. In cell experiments, ADAM15 gene silencing via siRNA knockdown decreased LPS-induced albumin permeability and neutrophil transmigration across endothelial monolayers [14]. Similarly, ADAM15 knockout or knockdown was shown to attenuate endothelial barrier injury and monocyte accumulation in vascular walls of ApoE deficient mice, hampering the progression of atherosclerotic lesions [12].

The mechanisms by which ADAM15 alters endothelial barrier properties are not well understood. Consistent with previous report that uncoupling or downregulation of cell-cell adherens junction proteins alters barrier integrity [47], our recent study suggests that ADAM15 promotes intercellular gap formation by inducing tyrosine phosphorylation of VE-cadherin and uncoupling of VE-cadherin/γ-catenin complexes at the cell-cell adherens junction [12]. This effect does not seem to depend on the extracellular domains of ADAM15 or its metalloprotease-based proteolytic function, but requires its cytoplasmic tail that is capable of binding and activating the Src family kinases for intracellular signal transduction. We reported that ADAM15 overexpression in endothelial cells did not induce or enhance junction protein shedding, and attempts to delete or block its metalloprotease domain failed to alter its effects on barrier function. In contrast, deletion of ADAM15 cytoplasmic tail, or blockage of its SH2 binding activity thereby inhibiting its signal transduction capability, effectively blocked ADAM15-induced endothelial hyperpermeability [12], [13]. This finding challenges the conventional paradigm of ADAM15 as a metalloprotease that acts by shedding or degrading junction proteins [4], [5], [48], [49]. In other words, it appears that the expression of ADAM15 (i.e., the presence of intact protein with full function in intracellular signaling via the SH2-binding cytoplasmic domain), rather than its enzymatic activity elicited by the metalloproteinase domain, plays a determinant role in regulating endothelial barrier structure and function. Therefore, strategies directed to inhibit ADAM15 expression via transcriptional regulation may prove effective in treating barrier injury.

MicroRNAs are small (∼22 nucleotide in length), endogenous noncoding RNAs that bind the 3′ UTR of target genes as complementary pairing, leading to mRNA degradation or translational repression [50]. Recent studies have shown that miRs are selectively regulated during different phases of inflammation where miRs suppress pro-inflammatory cytokines and chemokines, Toll-like receptors (TLRs), and transcription factors [51]–[53]. Administration of exogenous miR precursors attenuated lung injury in response to altered shear stress or inflammation [54]. miR-147, the murine homologue of human miR-147b, was induced by different TLR agonists, especially TLR4 which is the major receptor for LPS, and acted as a negative regulator of LPS-elicited signaling events in murine macrophages. Interestingly, inhibition of miR-147 significantly increased cytokine expression after TLR stimulation [53]. Although miR-147 has been identified as an anti-inflammatory miR in macrophages, there is no information available regarding its target and function in human vascular endothelial cells. Thus, a novel and unique aspect of our study is the identification of ADAM15 as a target of miR-147b in vascular endothelial cells with respect to barrier function regulation.

Our *in silico* analyses have identified many putative miR-binding sites in the 3′ UTR of ADAM15. Among them, miR-126, 147, 221, 222,17-5p, and let7 family are expressed in endothelial cells and interact with metalloproteases or tyrosine kinases [55]. Since miR-147b showed a high level of sequence complementarity to the ADAM15 3′ UTR, this miR became the focus of the current study. To verify targeting specificity, ADAM15 3′ UTR was amplified and cloned into a vector containing a luciferase reporter (pmirGLO/ADAM15). Endothelial cells were transfected with the reporter along with a scrambled miR or a miR-147b mimic, and the luciferase activity was measured. Transfection of human vascular endothelial cells with the miR-147b mimic decreased ADAM15 protein expression as quantitatively measured in Western blotting and visually observed under immunofluorescence microscopy. Compared to the level of reduction in total proteins, miR-147b-induced reduction in cell surface expression of ADAM15 was observed in a relatively small population of cells. This is not surprising, considering that ADAM15 is predominantly localized in the peri-nuclear region of the cytoplasm [56], and that we also observed a high level of intracellular staining (Fig. 3). Functionally, endothelial cells transfected with miR-147b mimic displayed an enhanced barrier function and blunted permeability response to albumin. Similarly, miR-147b mimic increased basal endothelial barrier resistance and attenuated LPS-induced barrier dysfunction. In contrast, transfection with miR-147b antagomirs increased albumin flux across endothelial monolayers, further supporting the barrier protective role of miR-147b.

It is noted that the current study focuses on manipulating ADAM15 expression via exogenous miR mimics or antagomirs and their effects on endothelial cell permeability. The in vivo impact of ADAM15 knockout in inflammation has been evaluated in several animal studies [12], [14], and the direct effects and Src-mediated signaling mechanisms of ADAM15-induced endothelial junction responses have been reported in our previous publications [13], [14]. Further studies are required to establish molecular mechanisms by which ADAM15 cytoplasmic domain recruits and activates Src as well as how Src alters junction or cytoskeletal proteins leading to barrier dysfunction. In this study, we chose not to repeat the signaling experiments but focus on the cellular effects of ADAM15-targeting miRs on endothelial barrier responses to septic agents. Since effective methods of organ-specific delivery of miRs have not been fully developed, and ADAM15-specific antagonists or drugs are not available, the current study is limited to in vitro cell experiments. The in vivo impact of ADAM15-targeting miRs will be evaluated in future studies.

In summary, the current study reports a novel function of miR-147b in regulating ADAM15 expression and protecting barrier function in human vascular endothelial cells. Our data suggest that targeting ADAM15 expression by exogenous miR-147b attenuates LPS-induced endothelial hyperpermeability.
